# Rapid Catalytic Template Searching as an Enzyme Function Prediction Procedure

**DOI:** 10.1371/journal.pone.0062535

**Published:** 2013-05-10

**Authors:** Jerome P. Nilmeier, Daniel A. Kirshner, Sergio E. Wong, Felice C. Lightstone

**Affiliations:** Biosciences and Biotechnology Division, Physical and Life Sciences Directorate, Lawrence Livermore National Laboratory, Livermore, California, United States of America; University of Edinburgh, United Kingdom

## Abstract

We present an enzyme protein function identification algorithm, Catalytic Site Identification (CatSId), based on identification of catalytic residues. The method is optimized for highly accurate template identification across a diverse template library and is also very efficient in regards to time and scalability of comparisons. The algorithm matches three-dimensional residue arrangements in a query protein to a library of manually annotated, catalytic residues – The Catalytic Site Atlas (CSA). Two main processes are involved. The first process is a rapid protein-to-template matching algorithm that scales quadratically with target protein size and linearly with template size. The second process incorporates a number of physical descriptors, including binding site predictions, in a logistic scoring procedure to re-score matches found in Process 1. This approach shows very good performance overall, with a Receiver-Operator-Characteristic Area Under Curve (AUC) of 0.971 for the training set evaluated. The procedure is able to process cofactors, ions, nonstandard residues, and point substitutions for residues and ions in a robust and integrated fashion. Sites with only two critical (catalytic) residues are challenging cases, resulting in AUCs of 0.9411 and 0.5413 for the training and test sets, respectively. The remaining sites show excellent performance with AUCs greater than 0.90 for both the training and test data on templates of size greater than two critical (catalytic) residues. The procedure has considerable promise for larger scale searches.

## Introduction

Given the success of the structural genomics efforts (1125 PDB entries) and many genome sequencing efforts, automated protein function annotation is now critical [1]. Annotation is the next step in turning the copious amounts of sequence and structural data into useful information in a biological context. At the core of many automated methods is the principle that sequence and structure dictate function. There are many perspectives and approaches to the application of this principle.

One approach is to infer function by focusing on global sequence or structural similarity. Global structural alignment procedures, e.g. LGA [2], PINTS [3], [4], and CE [3], [4], and sequence annotation approaches that indicate a structural or functional context, e.g. SCOP [5], CATH [6], GO [7], [8], or KEGG [9], successfully provide an enhanced annotation of the sequence of interest. In cases of high sequence similarity, sequence alignment methods such as BLAST [10] and CLUSTALW [11], [12] also enjoy wide success in inferring function in regimes of sequence similarity of >30% or more, with some high accuracy methods recommending 60% identity [1], [13]. Other approaches, which are largely phylogenetic in nature, include Hidden Markov Model (HMM) methods [14], [15], Evolutionary Trace [16], INTREPID [17], Phylofacts [18], [19], and Bayesian Monte Carlo inference [20]. Methods that combine sequence and structural information include EFICAz [21], [22], SOIPPA [23]–[25], DISCERN [26], PevoSOAR [27], and AnnoLite [28] and can provide improvements to sequence based methods alone. The success of global similarity-based techniques depends largely on the ability to distinguish conservation patterns that correspond to functional or catalytic portions of a protein sequence or structure. In general, enzymes tend to display motifs, or sites, which are very highly conserved in sequence and geometry, with the remainder of the protein displaying divergent global features, both in sequence and structure. The approach we present in this work is specifically designed to leverage the knowledge of specific catalytic site residues rather than to infer the functional features from global comparisons.

Other methods focus on those highly conserved regions associated with catalysis and biological function and have led to the development of protein function annotation algorithms that specifically focus on matching catalytic residue geometries. This more reduced description of a binding site led naturally to thinking of binding sites as a graphical object, consisting of nodes and edges that correspond to objects and distances (or other relations) between the objects. Graph comparison algorithms can then be developed that can rapidly locate a template pattern graph in a larger, target graph. This technique, known as the subgraph isomorphism problem, was originally proposed by Ullman [29] in a general context. Artymiuk *et al.* [30] appear to be the first to apply such a procedure to enzymatic site detection. Their work used the subgraph isomorphism procedure originally proposed by Ullman [29]. Later work by Artymiuk *et al.* expanded this approach beyond catalytic sites to other structural applications, such as the identification of tertiary structures [31], [32]. At the same time, Kleyvegt developed a site matching procedure originally designed to identify patterns in distance matrices determined by NOE peaks [33]. Later, Kleyvegt introduced a program called DEJAVU that detects protein motifs [34], using a depth-first searching procedure. This approach was later generalized to identify enzymatic sites with SPASM [35]. Other groups have used graph searching procedures, with specialized atom typing, such as Cavbase [36], PINTS [37], [38], and Query3D [39], or surface features as descriptors, such as eF-site [40], SuMo [41], and SiteEngine [42], whereby complex and physically motivated descriptors are used as nodes for the graph comparisons.

Other approaches to the template matching problem use a procedure known as geometric hashing [43], [44], which differs from the graph based approaches. Geometric hashing uses a cartesian grid to bin similar coordinates while graph based procedures use nodes and edges, independent of Cartesian coordinates. Geometric hashing is used widely in image processing and has been successfully adapted to structural approaches because it is well suited for comparing systems with incomplete feature sets, which require special handling in the graph comparison cases. However, geometric hashing is dependent on the frame of reference, and additional overhead is required to accomplish optimal translations and rotations for comparison. The Thornton group proposed a template matching procedure, named TESS [45], built on such a procedure. A later iteration, known as JESS [46] incorporated recursive ideas and threshold constraints to improve searching procedures. The JESS algorithm has been successfully incorporated as part of more comprehensive approaches to studying catalytic sites, such as the SABER method [47]. More recently, the Kavraki group developed a series of procedures built on a match augmentation procedure, MASH [48], that iteratively grows a template match from pairwise matches obtained through geometric hashing. Later developments from this group include the addition of residue hash matching, the LabelHash algorithm [49], [50], along with impressive optimizations at the hardware and software level to improve performance, and is among the fastest procedures reported. Other geometric hashing approaches include SitesBase [51], [52] and GIRAF [53]. The success of template matching methods led to the important recognition that a high quality database of enzymatic sites is needed. This recognition motivated the development of the Catalytic Site Atlas (CSA) [54], which is a manually curated table of enzymes and binding site residues and Enzyme Commission (EC) numbers [55]. The CSA, which we use in our work, has somewhat limited coverage of enzyme space, and the scale of such a database will always be strictly limited to the capacity of expert manual curators. As a result, many approaches have been developed which attempt to automatically locate structural features that may be used as templates. These approaches include physics based approaches [56], [57], statistical modeling of measures [58]–[60], dynamical considerations [61], and combinations of structural and sequence based measures. While all of these methods have met with varying degrees of success at interpolating known phenomena, there are always unexpected patterns of binding site architecture for which careful manual curation and characterization is needed. Other valuable resources related to this effort, including the MACiE database [62], the ProFunc [63] server, and metaservers like ProKnow [64], resulted from the success and utility of structure based approaches to understanding function. These approaches have not only been used to locate motifs in known structures, but also to design new motifs into proteins, as is done with SABER [47], which has opened new avenues to protein design [65].

The Babbitt and Gerlt groups have gone beyond matching catalytic residues to matching enzymes by their chemical mechanism. They established the concept of a mechanistically diverse superfamily, where the similarity among members is governed by the conservation of partial reactions within the protein family, rather than by sequence or structure conservation alone [66]–[68]. This approach is in contrast to a sequence based approach, which relies on global sequence similarity with the expectation that conservation patterns can point to residues of functional interest. It also presents an alternative to the Enzyme Commission (EC) based classification scheme [69], which builds a hierarchy based on the substrate reaction chemistry. This alternative approach to classification, with the emphasis on binding site architecture and conservation of partial reactivity, led to the development of the Structure Function Linkage Database SFLD [70], [71] and the Enzyme Function Initiative [68], [72]–[76], whose goal, among others, is to develop structural templates in a superfamily context.

We focus here on the optimization of a graph-based pattern matching algorithm, called the Catalytic Site Identification (CatSId), using catalytic residues templates as defined by the CSA. The CSA provides high quality and essential data for catalytic site studies and allows for a direct mapping of known structure to enzymatic function. The present procedure is optimized for best performance against a large library of diverse catalytic sites, as is present in the Catalytic Site Atlas. The input is a protein with unknown function, and the output is a list of candidate templates. The present work is designed to address the challenge of rapidly identifying motifs from a diverse catalog of sites, such as the CSA. Future work will address the issue of developing additional template libraries.

## Methods

**Figure 1:**
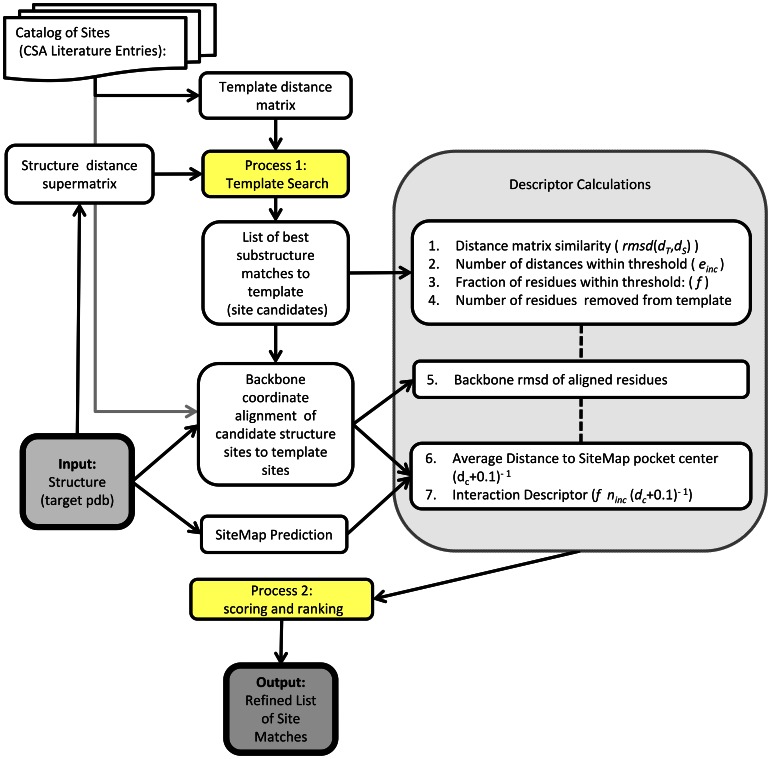
Flowchart for catalytic site search process. The main processes that were developed are highlighted in yellow. The structure (target PDB) is the input to the process. The initial template search stage is a fast search procedure that produces a set of site candidates. From this set, additional descriptors are calculated, including alignment binding site properties, which enhance the prediction quality through a logistic regression procedure.

Figure 1 describes the overall workflow, which takes a target (query) PDB structure and returns a list of catalytic sites that best match the target. The workflow contains two main *processes*, which are numbered and shown in yellow. Process 1 is a highly efficient template comparison routine that rapidly scans a library of catalytic sites geometries to generate an initial list of the best fitting sites for the template structure. Process 2 is a refinement procedure that quantitatively incorporates a number of structural features that are thought to be most descriptive of the binding sites. The coverage and quality of the search is primarily limited by the quality of the reference library of catalytic sites. We describe the preparation of this library in the next section.

### Construction of Site Catalog using Catalytic Site Atlas

**Figure 2:**
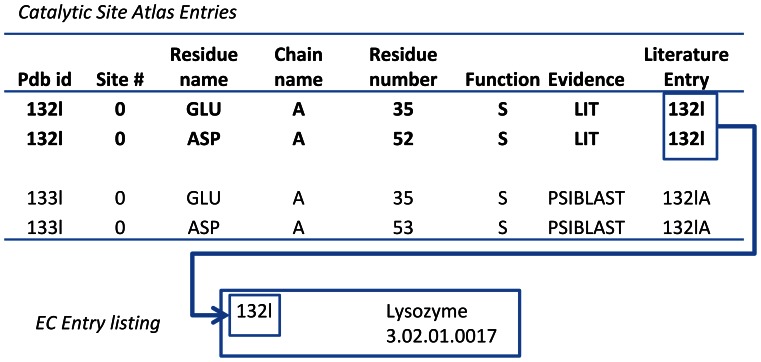
Diagram of Catalytic Site Atlas example entries and EC number lookup. Each site has a list of critical residues associated with it. Two entries associated with protein 132 l are listed. The literature based entry is shown in bold, and is what is used to populate the template catalog used for the present study. The EC number is listed in a separate table, and associated with the literature entry PDB id. The PSIBLAST entry shown is 1 of 45 PSIBLAST entries associated with 132 l.

The Catalytic Site Atlas [54], version 2.2.12, contains a list of manually curated catalytic sites based on published enzyme studies. Specifically, critical residues are listed for each binding site in the corresponding PDB structure. The complete Atlas also contains lists of related proteins based on sequence similarity. Figure 2 shows a diagram explaining the annotation for both literature based entries (LIT) and entries related through homology (PSIBLAST). Note, every PSIBLAST entry is associated with a particular LIT entry. While we use the PSIBLAST entries to identify possible amino acid substitutions (described in a later section), we use only the literature-curated entries as templates in the structural similarity comparisons.

**Figure 3:**
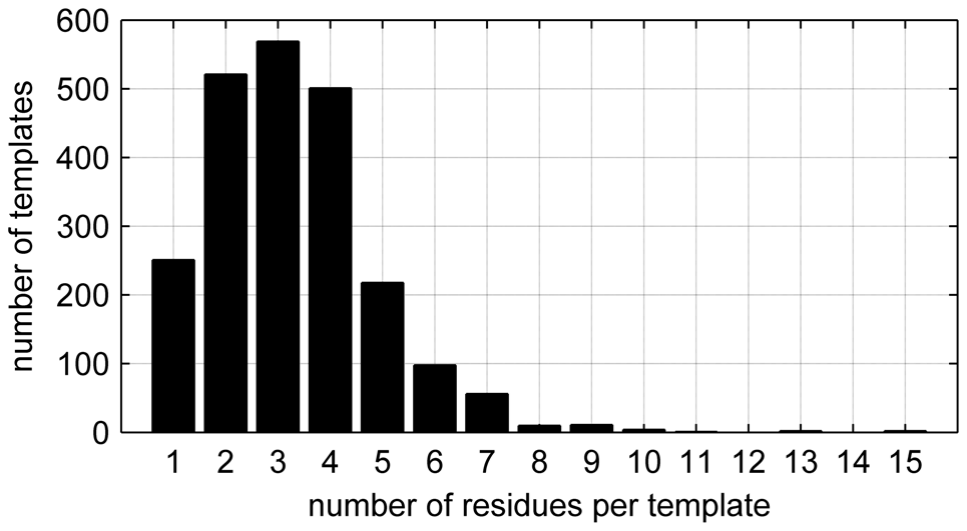
Distribution of template sizes for reannotated CSA.

From the table of literature-based templates, a residue-residue (Cα distance matrix is constructed for the critical residues identified by the CSA. The CSA was modified somewhat to account for curation errors, and additional annotations were included, as will be described. The resulting database has 736 unique EC numbers, each of which may have multiple PDB structures and multiple binding sites. In total, 2244 binding sites form the final database. Each of these sites can have multiple critical residues, and the distribution of template sizes is given in Figure 3. The majority of templates have 2, 3, and 4 residues, but templates as large as 15 residues are retained for the study; more than half of the sites contain either 3 or 4 residues. The single critical residue sites are kept in the table for completeness, but are not used in the present study, as the template matching procedure requires that there be at least two critical residues in the site. For each input (template/query) structure of interest, the template comparison routine performs a total of 1993 comparisons to the target structure, so that the template search process is the most time-critical operation.

### Annotations of Nonstandard Residues, Cofactors and Ions

**Figure 4:**
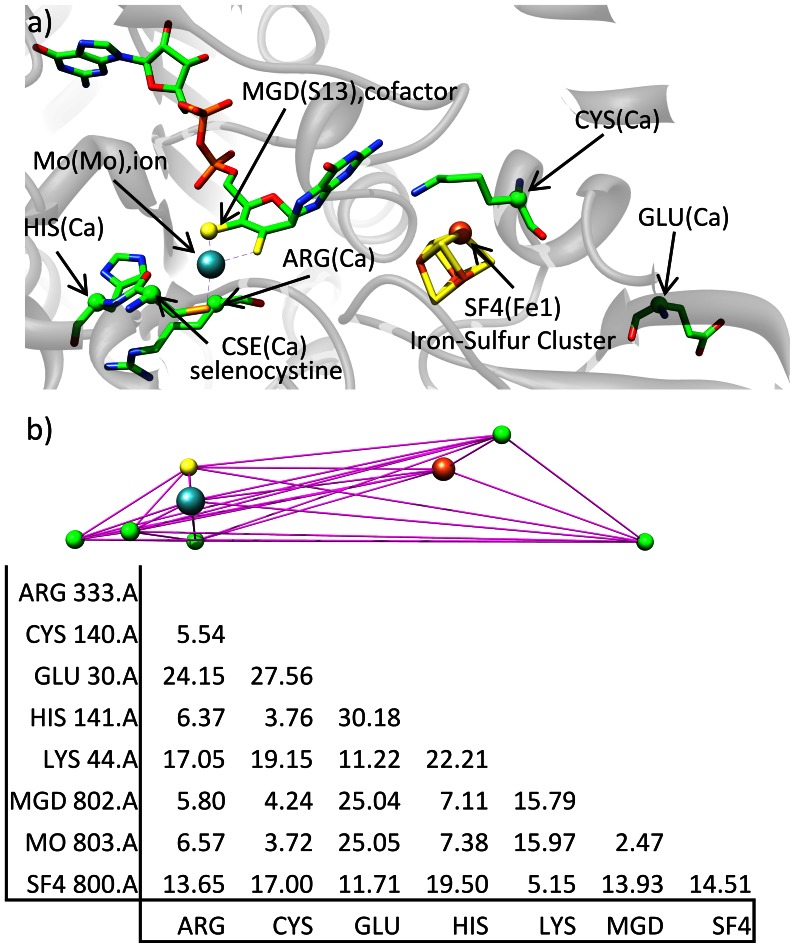
Example template distance matrix construction from PDB structure 1aa6, site 1 (E.C. 3.40.50.720). a) The CSA entry has a corresponding EC number, as well as a list of residues that comprise the site. Each residue has a centroid associated with it, which is shown in parentheses and represented as a balls in both a) and b). Cofactors and ions have a specific centroid assigned to them, while standard and nonstandard residues have the C_a_ as the centroid. B) a distance matrix with 28 elements is constructed from the resulting site coordinates and stored as for rapid comparison to a target structure.

Non-standard amino acids, ions and co-factors are also listed as catalytic residues in the CSA. To account for all of these additional groups in the CSA, we compiled a list of all of these molecules that require additional annotation. For the initial template matching algorithm, a unique Cartesian atomic coordinate is assigned to each residue. Figure 4 illustrates how the protein coordinates are transformed into a collection of atomic coordinates and, ultimately, a distance matrix for rapid comparison. While the template matching approach could be readily generalized to incorporate the notion of multiple atoms (or pseudoatoms) for each residue, the approach presented here uses a one-to-one mapping of each residue to a single atomic coordinate, typically that of the Cα-carbon. If the amino acid considered is nonstandard, it is assigned to have an equivalent standard amino acid identity. For example, in Figure 4, nonstandard amino acid CSE (selenocysteine) is treated as equivalent to a cysteine for all purposes. For both standard and nonstandard residues, the atomic coordinate of the Cα carbon of the residue is used. For ions, there is only one atom to consider, which is used directly for the coordinate. For cofactors, the relevant atoms were identified manually, based on either known reactivity sites or proximity to binding region. For example, the nitro group of the FAD molecule adjacent to the hydride donor group is identified as the donor. For the heme groups, the central iron is used. For the MGD cofactor in Figure 4, the sulfur (S13) closest to the molybdenum coordination site was selected, as its position is thought to be highly conserved.

### Additional Catalytic Site Atlas Annotations

We anticipate the need to locate a binding site in a monomeric target structure that may in fact be a member of a dimeric binding site. For example, the PDB ID 1fug protein forms a binding site in the interface between chains A and B. Trimeric binding sites are also observed in the CSA LIT entries.

Incidences where sites in the CSA are nestled at the interface of multimeric proteins are challenging to annotate properly. In these cases, all LIT sites within the CSA were thus analyzed to determine which sites were indeed located at a multichain interface. Sites containing anomalously large distances (>20 Å) were inspected manually. As an example of the annotation, a site 1xyz-0 (structure 1xyz, site 0) that occurs in a dimeric interface of chain A and B is relabeled as three sites: 1xyz-0cx for the original complex, and 1xyz-0A and 1xyz-0B for the portions of the site specific to chain A and B, respectively. Special care was taken to ensure that the chains and residues identified in the CSA corresponded to those identified in the PDB. There are also cases where there appear to be multiple, distinct binding sites within the same protein. Each such case is identified as a ‘multifunctional monomer’, or mfm, and each of the sites is identified by the chain and a unique index. For example, a multifunctional monomer with two sites on chain A would be labeled as 1xyz-0mfm, 1xyz-0A1, and 1xyz-0A2. Given that many input proteins are monomers with one putative catalytic site, this additional annotation helps to identify and filter anomalous cases.

### Allowance for Substitutions in Comparisons

The template comparison procedure, which is described in a later section, allows for residue identity substitutions when comparing binding sites by using a substitution matrix, much like a standard BLOSUM matrix [77], [78]. The essential difference in the present work is that the substitution matrix is specific to each template that is being compared. It also is of a binary form, which simply means that a substitution is allowed if the matrix entry has a value of 1, and not allowed if the entry has a value of zero. For all cases, forward and reverse substitution allowances are equivalent, such that the substitution matrices are symmetric.

**Figure 5:**
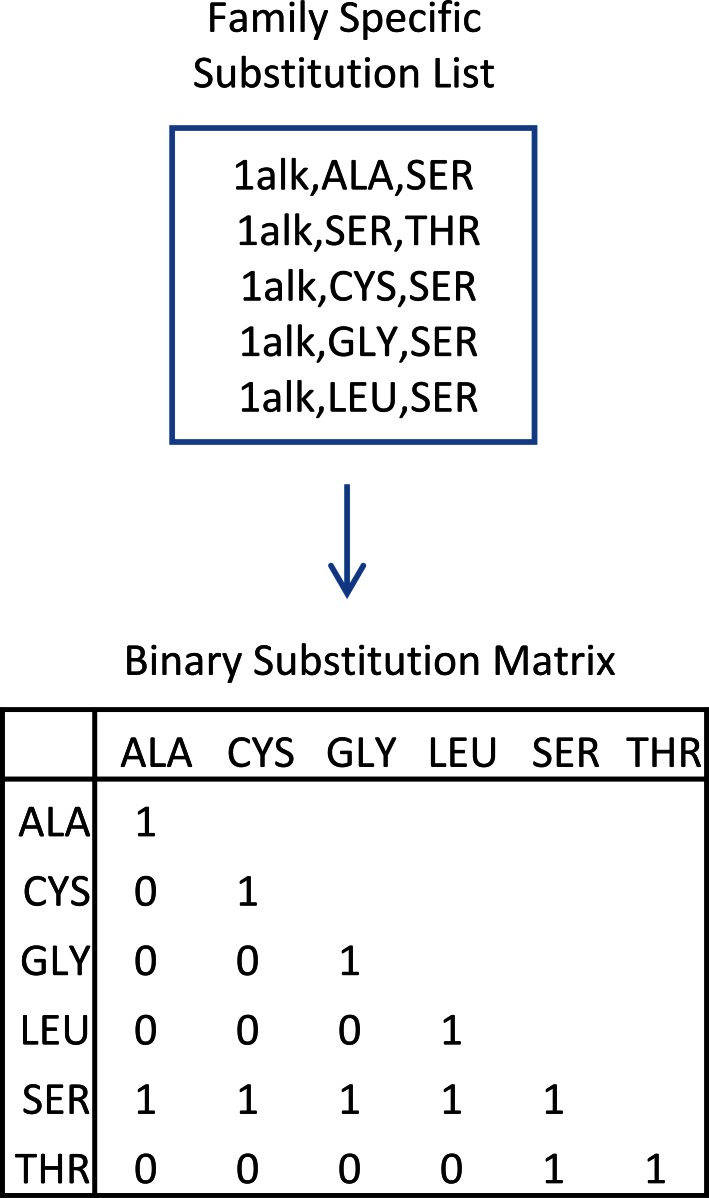
Template specific substitution matrix example. a) list of observed substitutions for the 1alk LIT entry family. All allowed substitutions are stored in a table (supplementary material) b) The family specific substitution matrix as constructed from entry in a) that is used as an input to template matching procedure.

The PSIBLAST entries in the CSA, while not used to construct the site library, are used to identify possible substitutions that can be allowed during the template matching procedure. To construct a substitution list, the PSIBLAST entries are screened for 2 criteria: 1) the number of residues in the PSIBLAST entry must match the number of standard residues in the LIT entry; and 2) the EC number as annotated in the PSIBLAST entry’s PDB header must match the complete four-digit EC number of the corresponding literature entry’s PDB header. Each PSIBLAST site is then compared to the literature entry site, and the observed substitutions are recorded in a tabulated form for each of the LIT entries. An example of observed substitutions for LIT entry 1alk as well as the resulting template specific matrix is shown in Figure 5.

**Figure 6:**
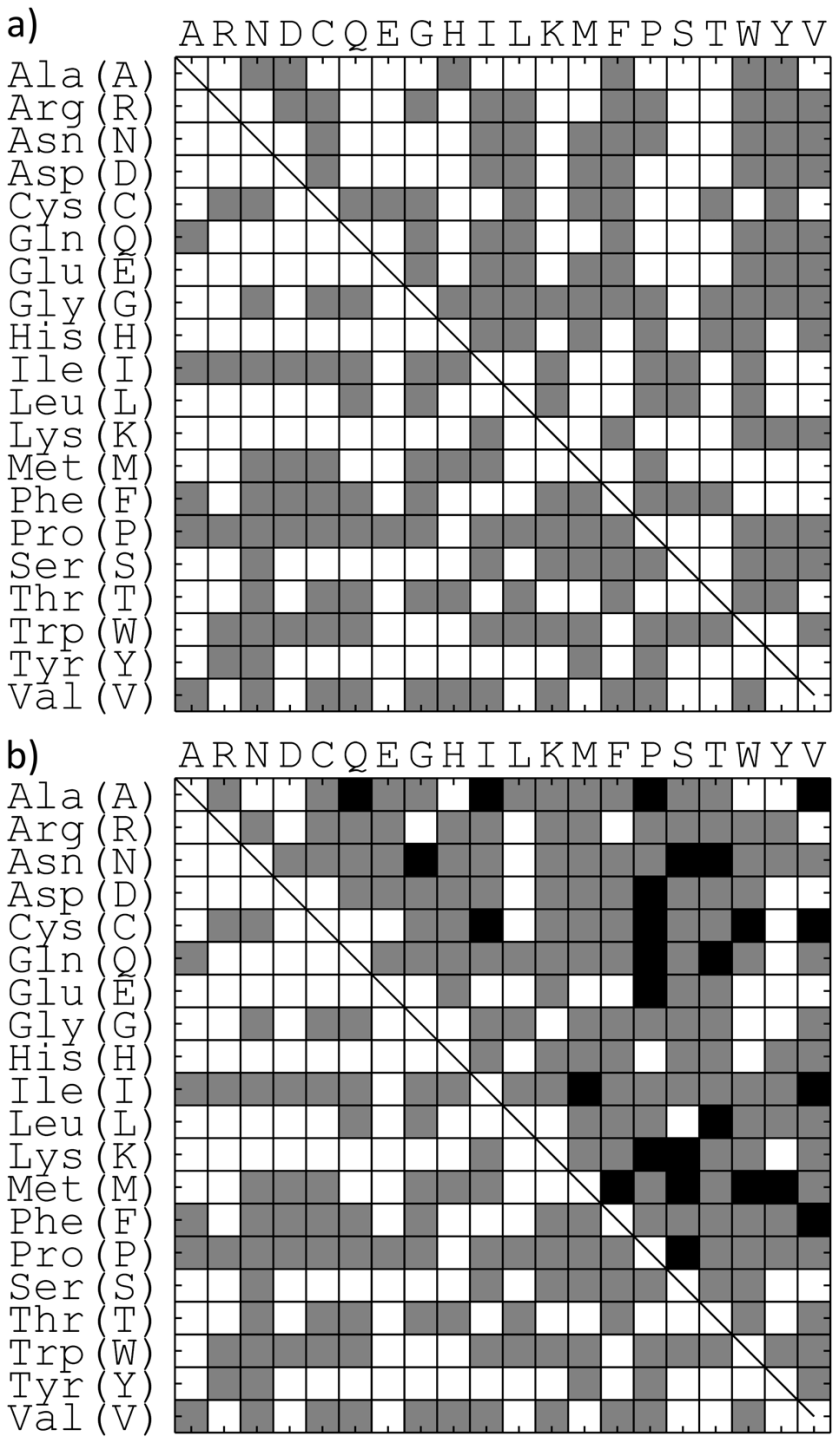
Summary of substitutions found in CSA dataset. a) Lower diagonal binary matrix indicates substitutions found in CSA matrix (gray if any substitutions are observed, white if none are observed), and upper diagonal is the binary form of BLOSUM 62 with gray indicated for values greater than −1 and white otherwise. B) Lower diagonal matrix identical to CSA binary matrix in a) for reference. Upper diagonal matrix shows differences between substitution matrices: (gray: no difference, black: CSA only, white: BLOSUM 62 only ).

Figures 6a and 6b (lower diagonals) show all observed substitutions in all of the templates studied. For comparison, the BLOSUM62 matrix is shown in Figure 6a (upper diagonal), which is modified such that a value greater than −1 is set to 1, and 0 otherwise. While this summary matrix is not used in the search, it is shown here for comparison.

**Figure 7:**
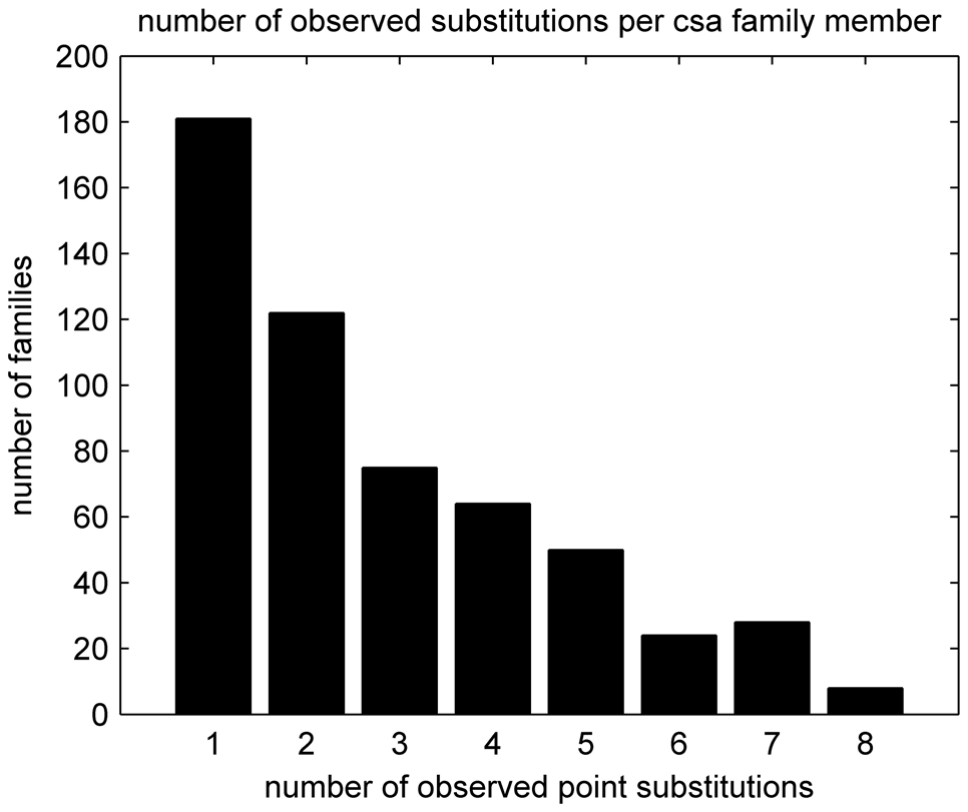
Distribution of number of substitutions per template. A total 302 family members (out of 967 total) display at least one substitution.

The full summary substitution matrix was found to be too general in most binding site comparisons, since each template family tends to favor a far more limited range of substitutions. Figure 7 shows the distribution of the number of observed point substitutions in the CSA. Out of 967 families, only 302 family members displayed substitutions. Of these 302 substitutions, more than half (181) are single point substitutions. The observed substitutions include those which are likely to conserve catalytic function, such as ASP→GLU, but also contain many substitutions that are not easily explained in a catalytic context, and appear to be family specific determinants of function. The substitution matrix procedure is used during the template search procedure (Process 1), which is described in the next section.

Cofactor substitutions were not considered in the present study. Ion substitutions were treated uniquely in that any ion can be substituted for any other ion, if it appears in the nonstandard list. This reflects the fact that ions of different identity often appear in a binding site as a result of experimental considerations. There are currently 22 allowed ion identities in the table.

All of the data tables described are available at http://catsid.llnl.gov.

#### Process 1: matching atomic distances using a template search procedure

**Figure 8:**
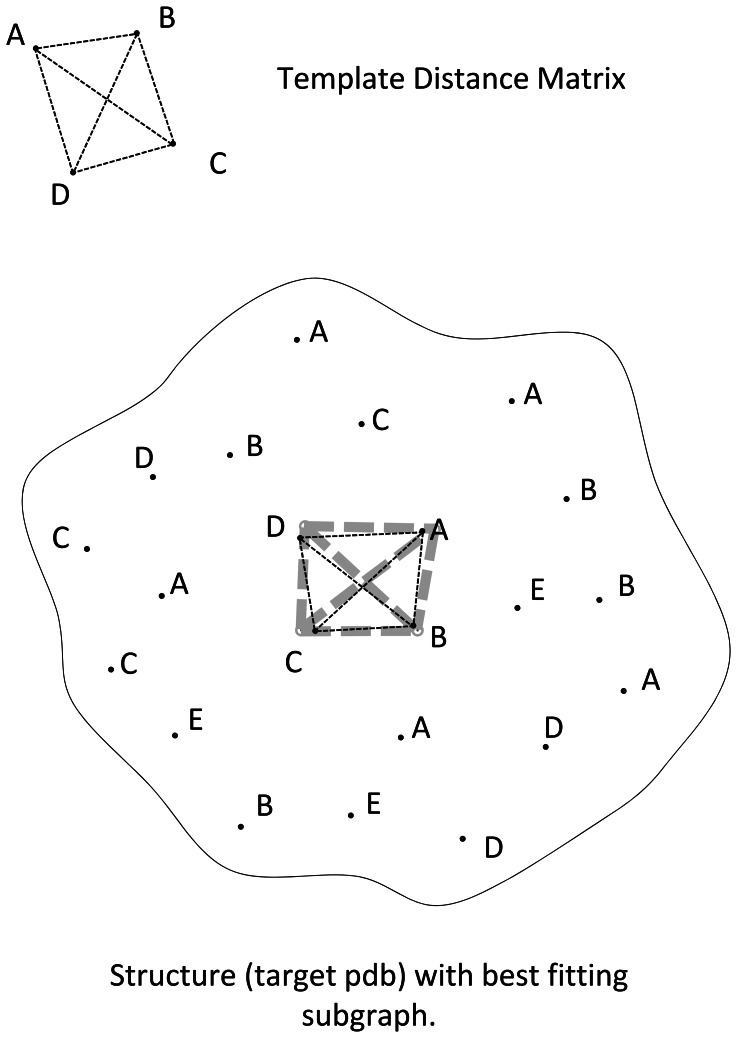
Diagram for template search procedure. The template distance matrix is shown for reference. The structure contains a supermatrix of distances, and the template search procedure searches for the sequence whose distance matrix best corresponds to the template. This is a variant of the Ullman subgraph isomorphism problem.

The basic search algorithm is designed to scan rapidly through a very large library of templates representing catalytic sites. As such, catalytic sites are described as a reduced set of coordinates – one coordinate for each critical residue, cofactor, or ion, as described above – in a distance matrix format (see Figure 4). Each of these distance matrices is identified as a template graph T. An example of a template with residue types A, B, C, and D is shown in Figure 8. For each target protein structure of interest to be studied, a distance matrix is computed for all atomic coordinates in the protein that have a residue (or an allowed substitution) that corresponds to one of the residues (including cofactors and ions) in the template structure. In the example shown, all residues of types A, B, C, or D would be included in the structure of interest (S) distance matrix calculation.

The general challenge, as illustrated in Figure 8, is to locate the subgraph in S that best matches template T. This is the subgraph isomorphism problem, first solved by Ullman [29], [31] The basic idea behind the subgraph isomorphism search is to systematically prune all possible subgraphs for which any edge does not meet a threshold requirement. The remaining list of allowed edges can then be used to construct all allowable candidate graphs. The problem is known to scale in nonpolynomial (NP) time with respect to the size of the subgraph to be compared. In many cases, however, careful pruning in the search procedure results in a more tractable scaling, as will be discussed. Ullman’s approach relies on the effectiveness of the threshold requirement to reduce the complexity. While this approach is vastly more efficient than an enumerative approach, it can still result in nonpolynomial scaling if the threshold requirement is too lenient. The approach presented here uses a similar *in situ* pruning technique, but adds additional screening and ranking procedures to further constrain the search space while maintaining a controllable degree of breadth to the search.

The procedure is described here briefly. We construct a list of sequences, or paths, through the structure protein. As this path is constructed, it is ranked according to the similarity to the template. For example, in the template given by Figure 8, we start by studying all sequences in the protein consisting of residue types A and B. As is done in the Ullman approach, we screen based on a distance threshold requirement. We found that a threshold of 1.5 Å allows for the detection of sufficiently similar catalytic sites. We then take the full list of eligible candidate sequences of residue types A and B, and compute the similarity between each constructed subgraph of the structure distance matrix and the template distance matrix formed by considering only A and B in the sequence. The top P_max_ scores are retained, where P_max_ is the maximum number of allowed paths per iteration. For each A-B sequence in the resulting list, the procedure is repeated when building a list of candidates with residue types A, B, and C. This additional screening process of keeping the top P_max_ scores at each step, rather than exploring all possible paths through allowed nodes, reduces the scaling substantially, as will be shown. Appendix S1 in File S1 provides pseudocode for this procedure.

### Scaling of Graph Search

The full subgraph search procedure scales in nonpolynomial (NP) time with regard to template size and is a large order polynomial with regard to target size. To see this, we compute the complexity of the problem as roughly corresponding to the number of distance matrix comparisons that are required during the buildup procedure. For a given target structure S, we compute the total number of possible candidate sites for comparison against the template T structure. This is given as
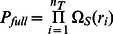
(1)Where 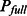 is the total number of nonredundant sequences that can be constructed with a sequence composition that matches the sequence of the target structure, 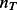 is the total number of sites in the template structure, and 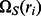 is the number of instances of residue type *r* (at template site *i*) that appears in the structure. As an example, Figure 8 shows a template with 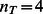 residues, and site 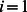 has residue type 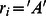, and this appears in the structure template 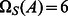 times. The number of times that the residue appears in the template affects the calculation. For the example in Figure 8, 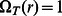 for all residues, but in general, the number of combinations is computed with the binomial coefficient,
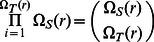
(2)which simply accounts for the fact that the resulting distance matrices constructed for the template comparison are invariant under the change of order when there are multiple residues with the same identity in the template. By default, all sequences are arranged first by alphabetical order and then by residue number. The total number of residues in the structure distance matrix is given as the sum over all unique residue counts in the distance matrix 
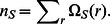


If we make a simple assumption that all sequences in the template are unique and the structure contains the same number of each sequence 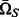 (assumed to scale with number of residues in the protein), then the number of distance matrix comparisons (neglecting any distance filter metric) would be 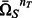, which is nonpolynomial with regard to the number of residues in the template distance matrix and a polynomial of order n_T_ with regard to target distance matrix size. Both scaling arguments are significant to consider (not just the NP scaling), as we are likely to encounter arbitrarily large target distance matrices, while our template libraries tend to be of a more limited size.

**Figure 9:**
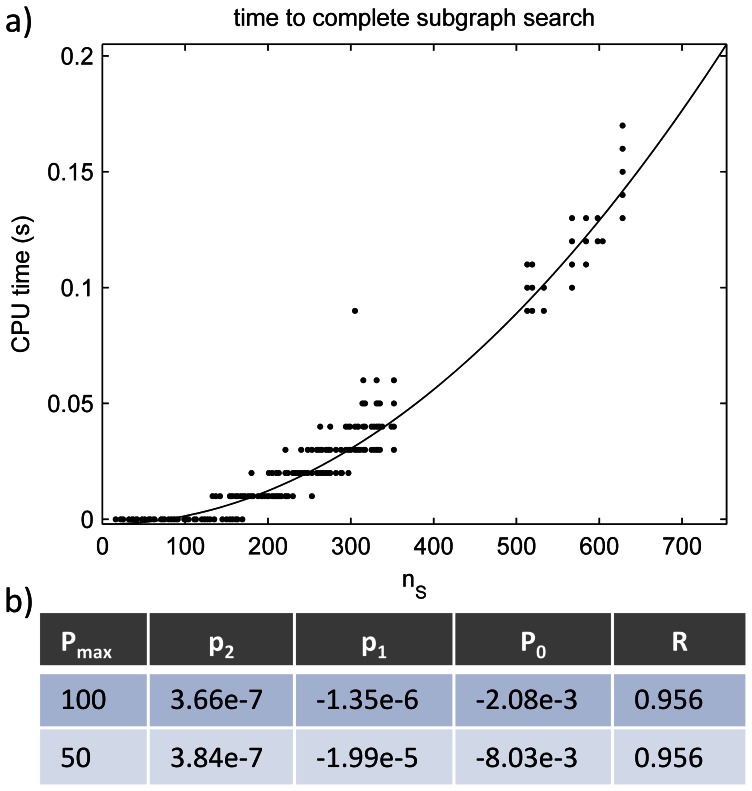
Typical calculation times for subgraph matching calculation. A) Data plot shown for P_max_ = 100. Timings are reported for pre-constructed distance matrices of protein 1 eus for comparison with 1980 templates from author curated CSA. n*_S_* is the number of sites in the distance supermatrix constructed for each template comparison. B) Polynomial coefficients of fits to data for P_max_ = 100 and 50, with correlation coefficients.

For the graph search, the scaling is vastly reduced, while still maintaining a user controlled variable to account for breadth in the search. The sequence is constructed iteratively, so subgraphs are constructed at each step to generate a distance matrix comparison to the template structure. If we disregard distance filters for the present discussion, the first set of subgraphs would contain the full complexity of the calculation, as given in Equation 1. Since only the top 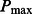 paths are retained, the total number of (partial and complete) distance matrix comparisons computed is
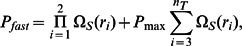
(3)and if we make the same assumption as above, we see that the scaling becomes 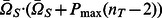, which is quadratic with regard to target size and linear in template size. A full discussion of the scaling properties using model systems is presented in Appendix S4 in File S1. Note, if 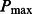 were set to the full number of possible paths at each node construction, then we would recover the NP scaling of the complete subgraph search. Figure 9 shows timings for searching for all templates (approximately 2000) against target distance matrices built from a representative protein, PDB ID 1 eus. Each reported time represents only the time to execute the actual subgraph search. While protein 1 eus has 356 residues in total, the size of the structure distance matrix varies with each template comparison, as the critical residue sequence composition is different for each template. There is considerable variability in both 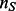 and 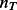 in this data set, but the overall scaling appears to conform to the predicted scaling behavior with regard to target size. Scaling with regard to target size is not as clearly visible for this dataset and is thus not shown. Data in Appendix S4 in File S1is provided to demonstrate scaling properties in a more controlled way, while this dataset represents actual expected behavior for the present template library. Typical times for this search are well under 50 ms when run on a single core, and even the largest of sites is easily completed under 200 ms. The scaling is clearly quadratic and nearly linear with respect to 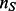. The quadratic fit produces a correlation coefficient of.956. Notably, the size of the template also affects the scaling, which is one source of variability. For example, the outlier data point at 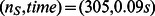 corresponds to a set of sites in family 2 sqc consisting of 15 template residues. The data plotted are for the setting of 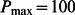, which was found to be sufficient to identify all templates (without losing the template in the pruning procedure) in both the training and test set studied. As is shown in Figure 2b, the influence of the value of 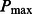 on the time scaling was found to be minimal. The present scaling properties point to the possibility of a vastly larger database of sites that could be readily searched.

#### Process 2: logistic regression to refine scoring

The graph search procedure (Process 1) used a minimal description of the binding site to facilitate a fast search. This representation, however, did not always unambiguously locate template matches, and further analysis was ultimately required, particularly for templates of 4 residues or less. The process of refining this initial list relies on a set of manually derived empirical approaches that were found to provide useful information about the binding site. These calculations are not as time intensive, but provide essential value added information that helps to select the correct binding site among a list of candidates.

While there are many variations to this approach, including many regularization strategies for a large number of descriptors, we use a standard procedure known as logistic regression, which relies on a sigmoidal multivariate indicator function to identify a result as being a positive match to the reference template. See Appendix S2 in File S1 for the definition of the logistic function as it is used in the present work. The parameters for this function fit using a maximum likelihood regression procedure, and the training set is described in detail in the Results and Discussion section. The descriptors were elucidated based on observations from the output of Process 1 and were selected based on goodness of fit. The descriptors that were considered are described in detail here.

### 1) Distance Matrix Similarity

The distance matrix similarity is computed directly from the site search procedure as the root mean squared difference between the template distance matrix and the Cα distance matrix constructed from the given sequence within the structure (rmsddm). The rmsddm is computed as:
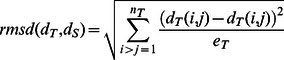
(4)where 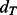 and 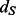 are the Cα distance matrices of the template and substructure (built from the target structure), respectively, and 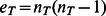 is the number of elements (or edges) in the target distance matrix consisting of 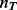 residues. It is a simple metric that is a direct output of the template matching procedure of Process 1.

### 2) Number of Distance Elements within a Threshold Value

During the comparison of the structure distance matrix to the template, each distance of the substructure, or edge, is compared to the corresponding template edge to determine whether it is within a certain inclusion threshold. If so, it is counted as an included edge. The current inclusion criterion setting is 0.5 Å. Note that this value is not the same as the screening threshold used in the template search procedure (1.5 Å) for the current study. The number of edges that meet this requirement is designated as 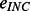. A related value that is more expressive of the number of corresponding residues within the inclusion threshold is developed in the next section. Note that descriptor 2 was used initially, but only as an interaction parameter in the final parameter set. The parameters used for the initial fit are given in Table 1 for completeness.

**Table 1:** Initial parameters are from a preliminary fit as described in Appendix S3 in File S1.

	Estimated coefficients by regression			
Descriptor	Initial	minimal	Inter-mediate	Final
**0. Intercept**	17.74	−3.09	−18.86	−4.8
**1. Distance similarity - rmsd(d_T_,d_S_)**	−2.1^a^	1.35^a^		–
2-residue templates	–	–	−0.60	0.21
3-residue templates	–	–	−0.92	−0.91
4–7-residue templates	–	–	−0.45	−0.01
**2. Number of distances within threshold**	0.94^a^	–	–	–
**3. Fraction of residues within threshold**	−18.18^a^	−0.84^a^	19.15	−1.73^a^
**4. Number of residues removed from template**	–	–	−2.764	–
**5. Backbone rmsd of aligned residues**	−3.94^a^	–		–
2-residue templates	–	–	0.49	0.55
3-residue templates	–	–	3.15	5.33
4–7-residue templates	–	–	0.50	1.84
**6. Average distance to SiteMap center**	−0.15a,b	–		–
1–3-residue templates	–	–		–
4–7-residue templates	–	–	−44.89^c^	18.61^c^
**7. Interaction parameter (** *f* ⋅ *n_inc_* ⋅ (*d_c_*+0.1)^−1^ **)**	–	–		–
2-residue templates	–	–	−3.80	2.83
3-residue templates	–	–	−11.47	−12.63
4–7-residue templates	–	–	14.81	3.18

The minimal parameter set is plotted as (f,C_a_) in Figure 7. Initial and intermediate parameter sets are used for preliminary rankings, as described in the text. Final parameters as used for remaining data analyses.

a Constrained to be equal across 2, 3, and 4–7-residue templates.

b Computed as *d_C._*

c Computed as (*d_C_*+0.1)^−1.^

### 3) Fraction of Correctly Placed Residues

The goal is to develop a metric that estimates the number of residues that are considered to be included in the template by counting the number of distances that fall within a certain threshold. In order to do this, we derive the relation between the number of edges that fall within a nominal tolerance (0.5 Å, as above) and the corresponding number of nodes that are correctly placed. We consider a template having 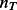 residues, or nodes, and the search-result graph of the corresponding structure residues having 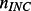 nodes that are considered to be within tolerance and 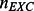 nodes that are considered to be outlying residues. The total expression is.
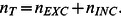
(5)


The total number of edges can be written, correspondingly, as
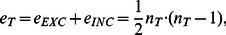
(6)where 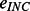 is measured directly from the search procedure. For a graph of 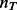 nodes, we assume that 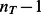 edges are lost by removal of one node, 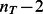 by removal of the second node, and so on. We count the number of excluded edges as



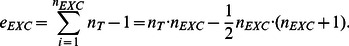
(7)Substituting Equation 7 into Equation 6 gives
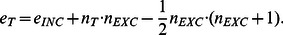
(8)


Solving Equation 8 for the number of excluded nodes is a quadratic equation. Taking only the physically meaningful root gives
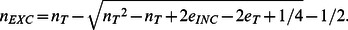
(9)


This expression has a minimum value of 0 when all edges are within the cutoff threshold and a maximum of 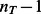 when no edges are within the threshold, since the final node is not counted in the exclusion.

In either case, we can estimate the fraction of residues that are included by the simple form given as
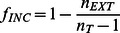
(10)


### 4) Number of Residues Removed from Target

There are many cases where a residue that is part of the template sequence is not present in the target structure. Rather than exclude these from the search altogether, the method used here is to construct a template with a sequence with only those residues that appear within the structure. Accounting for missing residues in the analysis is particularly important for Process 1 to work correctly. If a residue is entirely absent from the target, the graph search procedure rebuilds the template with the residue removed so that the subgraph search works correctly. The removed residue can be either standard or nonstandard. The number of missing standard residues is recorded as a descriptor. For example, if the structure in Figure 8 contained no ‘D’ residues, the procedure would be to construct the template with only ‘ABC’ residues and locate the best fit to the ABC graph. The calculation of Eq. 10 would be then recorded as 1.0, but the number of missing standard residues would be recorded as 1 if D were a standard residue, and 0 if it were a cofactor or ion. While this descriptor was not ultimately used in the final parameter set, it was used in an intermediate parameter set and is reported here for completeness.

### 5) Backbone RMSD of Aligned Residues

Once a substructure is identified, an alignment can be performed. For all standard and nonstandard residues, the atomic coordinates are selected from the backbone of the template and structure proteins, which includes the N, Cα, and carbonyl carbons of the residues. For cofactors and ions, only the centroid atom is used for the alignment coordinates. Since the atoms to be aligned are always in a one-to-one mapping, a least-squares quaternion alignment procedure [79]–[81] is used to align the atoms and compute the root mean square distance between the aligned coordinates. This metric is intended to capture more structural information about the relation between the binding sites, since the Cα metric is a simpler metric designed to facilitate the library search procedure.

### 6) Average Distance to SiteMap Pocket Center

The Schrodinger SiteMap package [56], [57] is used to generate a list of binding pocket candidates which are rank ordered according to physical properties that determine the druggability of the pocket. All pockets that are identified are considered. The SiteMap procedure is very reliable at identifying protein surface concavities, or pockets, that often correspond to the catalytic center. There are some exceptions in the CSA that do not conform to this model, however. Trypsin, for example, has catalytic residues on the protein surface to facilitate cleavage of a protein upon forming a protein-protein interaction. In general, however, a catalytic region is thought to exist within what is typically identified as a pocket, and the sitemap prediction provides valuable additional information about the binding site of interest. It is also based entirely on physical properties alone and is completely independent of any information-based approach.

The center of each pocket identified by SiteMap is computed from the simple average of the coordinates of the ‘gridpoints’ that populate each pocket. The average distance to the center is then computed as the average of each of the distances from the residue centroid coordinates to the pocket center and recorded as 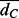. The closest average distance is selected as the descriptor for the binding site candidate, and 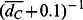 is computed so that the metric is larger for more favorable distances, with the constant of 0.1 added to the denominator to prevent singularities.

### 7) Interaction Descriptor: Number of Correctly Placed Residues and Site Center Distance

The use of interaction descriptors is a standard practice in logistic regression [82]. For the present case, the descriptor originates from the observation that a favorable configuration around the binding site is more significant if there are more residues in the binding site described. Many of the descriptors are intrinsic variables, as they are normalized to either the number of residues or the number of edges in the related graph. All of the descriptors listed are intrinsic except Descriptor 2, which is extrinsic with regard to the number of edges 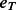. However, Descriptor 2 is not used as a final parameter (see Table 1). To provide another extrinsic descriptor, the quantity 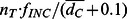, is used, which combines Descriptors 3 and 7.

### Size Specific Parameterization of Descriptors

The size of the template site affected the character of the site search and characterization, particularly for templates with fewer than 4 residues. For example, a site with few residues will produce predictions of a lower certainty from the template matching procedure, simply because there is less information in the distance matrix of smaller templates. As a result, the parameterization of many of the descriptors was treated as being dependent on the number of residues in the template. To achieve this, a Heaviside-like switching function was used that allowed for different parameters to be fit for templates having different numbers of residues. The descriptors for which this appeared to provide improvements were 1, 4, 5, and 6. For all cases given except descriptor 6, the residue-dependence was parsed as template sizes of 2, 3, and 4–7 residues. Table 1 lists all parameters and the fitted values from an initial scoring function, a “minimal” regression that illustrates the discriminatory value of a limited descriptor set, and the final logistic scoring function used in this study. The total time to completion for the full analysis, including SiteMap and all postprocessing, was under an hour running on one compute node, comprising of 16 compute cores (2.3 GHz AMD Opterons) and 32 Gb of shared RAM. This runtime includes additional overheads that were not specifically optimized, as the focus here was in optimizing the core routine.

## Results

We developed an automated protein function identification method based on the hypothesis that catalytic residues and their geometric arrangement are key determinants for enzymatic chemical activity. Our approach transfers function to a query protein structure if the query structure displays amino acid positions and identities consistent with structures of experimentally annotated enzymes. The procedure performs geometric matching of putative catalytic residues on a query structure to a library of experimentally annotated catalytic residue positions. The reference structures library came from the original CSA (Table of Thornton version 2.12.12), as is found on the Thornton site http://www.ebi.ac.uk/thornton-srv/databases/CSA/. In addition, we use other descriptors, based on both geometric and physical properties, to improve the predictions and use these descriptors in a logistic regression scoring procedure. Table 1 lists these descriptors, which include a variety of geometric descriptions and quantifications of both the reduced representation of the binding sites and the full backbone coordinates of the binding sites. The average SiteMap distance, which is a more physical descriptor, is also included.

### Selection of the Training Set

As mentioned earlier, the CSA contains two categories of entries, consisting of literature verified entries (labeled as LIT) and PSIBLAST labeled entries, where each PSIBLAST entry is related to a LIT entry via sequence homology. For the training set, only PSIBLAST entries were selected as inputs for the search procedure. In this exercise, the input structures play the role of query sequences. A “correct” search result (true positive) is defined as a template whose four digit EC number matches the target EC number as it appears in the protein databank file.

To assemble the training data set, 108 CSA PSIBLAST entries were randomly chosen from the CSA. This list was further down-selected by eliminating:

- Targets (remaining PSIBLAST entries) that did not have the same full four-digit EC number – as given in the protein databank file – the corresponding LIT entry.
- Targets that contain only one residue in the binding site or that have a related LIT entry with only one residue. Single-residue entries are retained in the CSA literature table for completeness, but such templates are not used in the search procedure, as they have no distance matrix.
- Targets for which there was no search procedure result for the related LIT template. This can occur when one of the residues to be compared falls outside of the 1.5 Å threshold, or when the correct substructure does not fall within the top 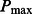 scores in the search procedure at any step in the iteration. The original setting of 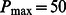 was later adjusted to have a value of 100 to address this limitation.

After applying these criteria, 66 structures remained. The list of PDB codes in the training and test data sets are given in Table 2.

**Table 2:** List of PDB codes used in test and training sets.

Training Set
1b7g,1bwk,1cla,1cy1,1d6n,1dbz,1dv7,1f4c,1g02,1ge6,1gin,1gzg,1hqd,1hzz,1i2o,1igw,1jiu,1jp7,1kgq,1krc,1l5w,1lmz,1mj5,1nwc,1ojp,1p5g,1q2e,1rrj,1rsm,1rwp,1s70,1t2a,1t3z,1t4d,1ucl,1w23,1w3y,1wkl,1wo8,1wow,1wyi,1yai,1ykn,1ytn,1z83,1z8x,2aad,2bcd,2be7,2brv,2g22,2hb1,2ido,2j4s,2ori,2p9e,2qll,2qmo,2v6s,2vel,2vf5,2vmn,3c80,3cn9,3cuf,3d4z,3dt2,3dzc,3eju,3fpd,3gxf,3hh4,3i4c, 3i9l,4fua
**Test Set**
1ado,1ajb,1blm,1cib,1d4e,1e2r,1ep9,1eus,1f3x,1f49,1fdv,1g1y,1g87,1ggf,1i45,1ib4,1iu8,1jol,1k3t,1kak,1kg4,1khn,1kvy,1l7a,1nto,1nwr,1p07,1rry,1ru1,1tsl,1wdd,1xpt,1xv8,1xww,1yja,2a3t,2ayl,2ayo,2c0h,2cba,2cnh,2ewn,2ez9,2fbp,2fpt,2nu8,2nze,2o3q,2otc,2pov,2ppy,2qd4,2qu9,2veg,2wfp,2whr,2zj3,2zyd,3bbf,3c52,3czn,3dhe,3ehb,3fgd,3gtd,3it1,3pf

### Building the Logistic Regression Model based on the Training Set

The search procedure (Process 1) was performed for each of these target structures. The results for each structure – that is, template “hits” – were ranked by their score based on an initial, exploratory logistic regression run on a more limited data set (see Table 1 for initial parameters). To ensure that “difficult” cases are present in the training data set, the top 20 hits were retained for each of the 66 PSIBLAST targets. We searched for correct results within the top 20 hits for each template. In cases where the correct template was not among the top 20 hits, the correct hit was added to the data set. During the course of estimating potential scoring functions, tests were conducted on the effects of rescoring and re-ranking all of the hits for selected targets. These tests showed that the training sample hits did not sufficiently represent the universe of negative cases. To remedy this, additional negative cases were randomly selected from the search procedure result and included in addition to the top 20 hits as described above.

The training set, comprised of this list of data points, descriptors, and positive/negative annotations, was provided as an input to the logistic regression procedure in the R statistical package [83]. A series of parameter combinations were tried, including different combinations of template size dependence. This process was guided generally by selecting combinations of parameters that lower Akaike Information Criterion (AIC) and which ultimately result in a high value of the AUC (for the training set alone). Table 1 lists the final combinations of parameters that were deemed to give best performance overall.

### Validation of the Model against a Test Set

The test set was constructed in a similar manner to the training set. From the CSA, 106 PSIBLAST entries were randomly selected, excluding those which were templates, single-residue sites, or which did not have matching EC entries in the PDB record, as was previously described. In addition two entries that had very poor structural similarity to the related LIT entry were also removed from the test set. One of these two removed entries was a member of the 1 gim family, 1 hop, which has rmsddm of 3.25 Å compared to the 1 gim template, and a member of the PDB ID 1fr8 family, 1j8w, which has rmsddm of 2.15 Å compared to the 1fr8 template. The final test set contains 67 PSIBLAST entries as targets.

To select a list of known negatives and positives, we used an intermediate iteration parameter set to generate scores and rankings. The parameter set is in Table 1 and labeled as an intermediate. This parameter set was ultimately refined for performance, but the test set data, did not change during this process. As was the case in the training set, the top 20 hits against each target were selected, with positives not in the top 20 included in the analysis.

To assess the quality of both the training set and the test set, a standard Receiver Operating Curve (ROC) analysis was performed, with the area under the curve (AUC) treated as a quality metric. See Appendix S2 in File S1 for a definition of the statistical terms used. In general, a ‘perfect’ ROC curve would be a step function, and the area under the ROC (AUC) would be 1. By comparison, a ROC plot that falls on the diagonal would be considered no better than random with an AUC of 1/2.

Before the receiver-operator characteristic (ROC) curves were constructed, “duplicate results” were deleted from the test data set. That is, occasionally there were multiple correct hits (“positives”) – typically, additional binding sites on a multimeric template. Similarly, there were multiple incorrect hits (“negatives”). We did not want to overstate either the true positive rate or the false positive rate in constructing the ROC curves, so only the highest-scoring of such duplicate results were retained in the test data set.

**Figure 10:**
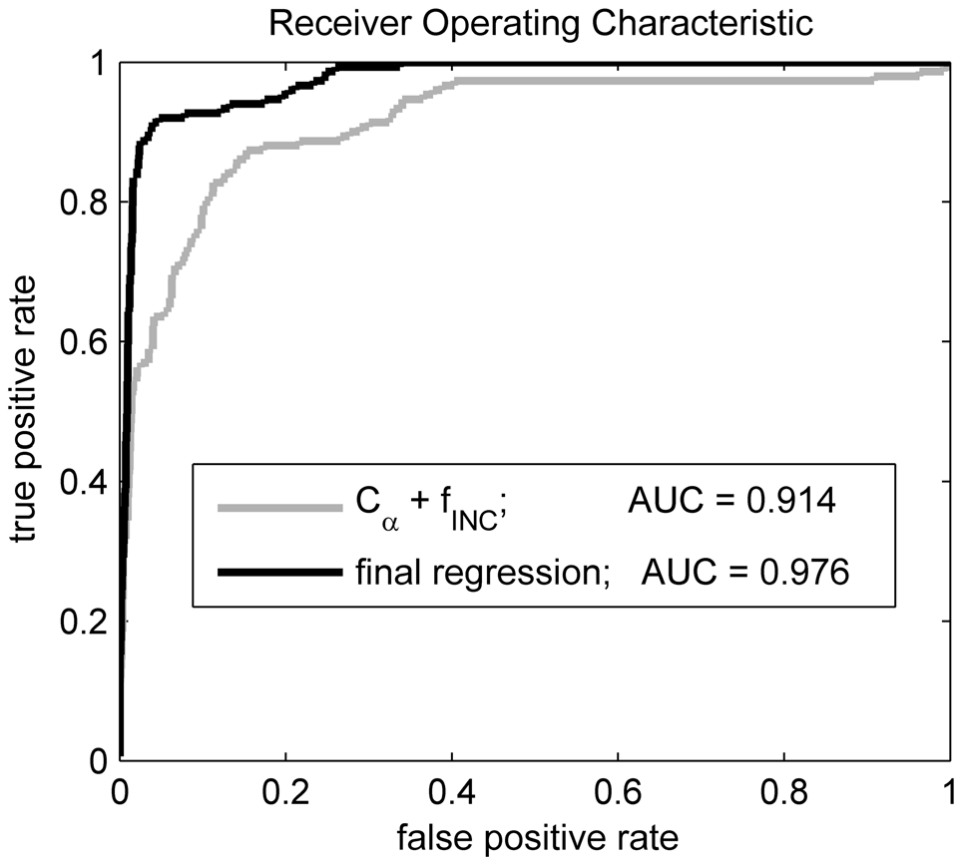
Comparison of performance on training data using minimal (naïve) descriptor set and final parameter set. Curve in gray is generated using descriptors resulting from analysis of process 1 output only. Final regression contains full descriptor set as described in the text.

To judge the improvement due to adding the various descriptors, a regression was performed using a minimal parameter set, which corresponds roughly to the information provided by Process 1 alone. The AUC is vastly improved from 0.877 to 0.971, when the final parameter set is included (Figure 10). Figure 10 clearly shows that the descriptors improve the predictive capacity of the procedure. Thornton also reported a multistage procedure, with the second stage consisting of a statistical procedure [46] but with a less comprehensive descriptor set, and we were able to achieve higher AUCs.

**Figure 11:**
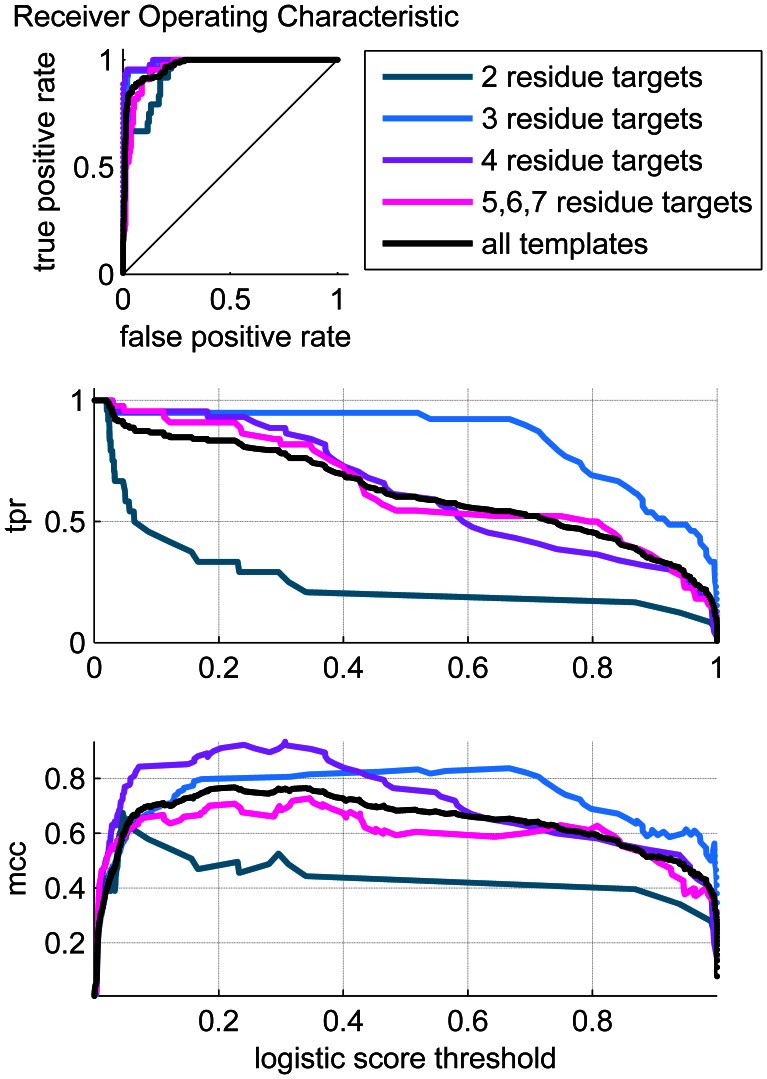
Performance of training dataset. a) ROC plots b) True Positive Rate (TPR) vs. logistic score (threshold) c) Matthews Correlation Coefficient versus logistic score.

**Figure 12:**
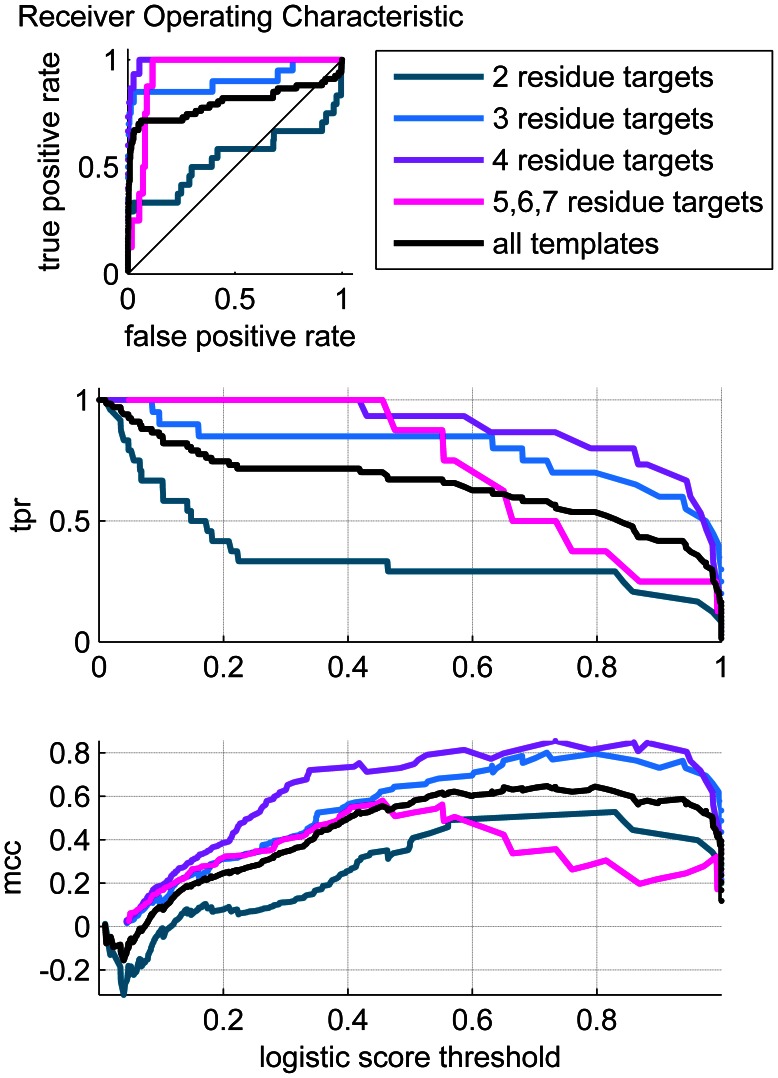
Performance of test dataset. a) ROC plots b) True Positive Rate (TPR) vs. logistic score (threshold) c) Matthews Correlation Coefficient vs logistic score.

The overall performance of the regression model is shown for both the training set (Figure 11) and the test set (Figure 12). Figures 11a and 12a show the ROC plots with the overall performance plotted in black. The AUCs for the complete training and test set are 0.9714 and 0.8951, respectively, which is very good performance overall, both in terms of the quality of the fitting to the training set, as well as its ability to correctly predict the test data points.

## Discussion

Our main interest here is to evaluate the performance of our template identification approach. A unique quality of our procedure is that it is able to detect binding sites with a diverse template library (the CSA). Particular attention was given to a procedure that could identify templates of varying size. To better understand the influence of binding site size on the ability to correctly locate the template within the targets, the performance was partitioned by the size of the template corresponding to the particular target studied. The performance dependence on binding site size is most evident in Table 3. We note first that the two-critical-residue cases perform poorly on the test data. The two-residue cases are extraordinarily difficult to detect in general because there are many false positives particularly in the graph searches. The three-residue cases are difficult to detect using the template matching algorithm alone. For these cases, the logistic regression procedure provides a markedly better performance. Since catalytic triads, or sites consisting of three critical residues, make up such a large component of the CSA (roughly one-quarter of the CSA are triads), the addition of the postprocessing steps provide good performance on what would otherwise be considered as marginal performance cases. The choice of descriptors for the second stage scoring also provided a significant improvement in performance. Figure 10 shows the improved AUCs obtained by the incorporation of the final, optimized descriptor set relative to a minimal descriptor set.

**Table 3:** Area under ROC curves (AUR) for templates of differing sizes, as well as full dataset.

	AUC(train)	AUC(test)
*n_T_* = 2	0.9411	0.5413
*n_T_* = 3	0.9821	0.9040
*n_T_* = 4	0.9932	0.9935
*n_T_* = 5,6,7	0.9622	0.9369
All	0.9714	0.7989

The level of sensitivity is difficult to directly compare between our algorithm and those which are widely used. The original JESS development study [84] provides some statistical measures that can be compared. The dataset used by Torrance *et al* excluded a larger fraction of the CSA than our present study. The Torrance dataset [84] included only families with at least three catalytic residues, and family members (PSIBLAST entries) that did not have residue substitutions compared to the family parent (LIT entry). We developed a similar dataset (listed in Table S1) for a more direct comparison. With this dataset, our overall AUC is improved from 0.976 (see Figure 10) to 0.981 (see Figure S1a). The Torrance study does not report AUCs, but they do report MCCs and corresponding sensitivities, or TPRs, which can also be regarded as fitness measures. For their best template matching approach, they report a maximum MCC of 0.84 and a TPR of 0.75. Our broader dataset study reports an MCC of 0.75 with a TPR of 0.75. The MCC is a measure of efficiency in distinguishing positives from negatives, while the TPR is the fraction of correctly identified positives. While our MCC is less than the Torrance measure, our TPR is comparable. Most importantly, the metrics measure performance relative to the dataset studied. Since our dataset includes more challenging cases, the difference in performance is not entirely unexpected. The performance of the training set on two residue site templates alone can explain this discrepancy. Figure 11 shows that the MCC of the two residue site set peaks at 0.65, giving a TPR of only 0.6, which is substantially lower than any of the other templates considered. When we compare the performance of our approach on a dataset that more closely follows the Torrance study, we obtain a broad maximum MCC of better than 0.80 with TPRs ranging from 0.84–0.92 (see Figure S1b). The MCC values here are comparable, and our TPRs are clearly better. We also note that some of the earlier work from Wallace [85], when using JESS, reported an AUC of 0.82, as compared to our challenging training set AUC of 0.976 (Figure 10). The Kavraki group reports AUCs that are similar to ours [4949] but also uses a small subset of the CSA, excluding three residue site templates. To our knowledge, our study uses one of the most comprehensive libraries of sites for training and testing. Our first generation performance measures are comparable to the work of the Thornton and Kavraki groups, which are representative of the state of the art in catalytic template matching.

Catalytic sites involving four or more critical residues can often be unambiguously identified using graph comparison results, procedure, and the addition of the descriptor procedure provides an additional enhancement. The performance in identifying sites of this size range is likely to be affected by the size of the dataset concomitant fitting procedures, such that it may be a guideline for constructing future catalytic annotations to include at least four or more critical residues as a catalytic site definition. For most cases, the true positive rate rises to a value close to 1 for logistic thresholds greater than 0.75, suggesting that a threshold value of approximately 0.75 is a good indicator of a positive. Thus, we can determine the quality of the match not only by the ranking, but by the value of the scoring function relative to the threshold. Sites of four residues provide high quality matches and do not present scaling challenges. Thus, the four residue sites could be regarded as an ideal template size for the purposes of designing template libraries.

For larger templates (5–15), more pressure is placed on a scalable search than on the false positive rates. The number of coordinates in the template is sufficient to define the site uniquely, but the large number of possible comparisons to construct can result in NP scaling. Our approach has been shown to scale linearly with template size, which allows for a nearly arbitrarily large template to be compared with relatively modest computing resources.

We believe the method has great promise for scalability because of the very efficient graph search algorithm presented. The postprocessing steps provide an additional increase in performance, and the overall procedure is very robust with regard to template size as a result. Future work will incorporate searches across the entire protein databank.

### Conclusions

We have developed an automated procedure for protein function prediction based on the identification of catalytic site residues, called the Catalytic Site Identification (CatSId). The procedure hinges on matching residue arrangements in a query series and comparing (matching) to a library of manually curated catalytic residue templates. The initial template matching procedure is an extremely rapid subgraph search method, while not sacrificing the necessary breadth in the search to locate all candidate sites. The scaling properties of this template search procedure suggest that much larger libraries of catalytic sites can be searched readily without catastrophic limitations on library or template size. Since the template matching procedure has excellent scaling properties, the overall procedure will be able to very accurately identify templates with a larger number of critical residues without scaling difficulties. The logistic regression procedure provides improvement over the template matching procedure alone by incorporating a number of empirically derived descriptors to enhance the prediction. The success of this approach provides a foundation for many structural approaches to enzyme functional annotation. For example, a particular enzyme motif can now be readily used as a template to search against the entire protein databank for viable protein structures that may or may not be annotated. The detection procedure does not rely on global structural features in the way that many structural similarity procedures do and could open new directions in enzyme annotation and design. As a general procedure, however, the site identification procedure is of course strongly limited by the quality and quantity of the catalytic site library. While the CSA is an invaluable resource, a significantly expanded database of catalytic sites is clearly needed to advance the field. The scaling of the search of an expanded library is easily within current computing capacity. The performance of the present approach is sufficiently impressive as to recommend the development of a larger database in the future.

## Supporting Information

Figure S1 **Performance of dataset for comparison to Torrance study.** A) Receiver Operating Characteristic b) Sensitivity (TPR), Predictive Accuracy and MCC.
(TIF)

Figure S2 **Timings for synthetic template comparisons.** Data are shown as points with solid lines as least squares fits.
(TIF)

Table S1 **Targets (member of a template family) and templates – identified by PDB code – included in comparison with JESS.** Where the template is not present the algorithm did not return a correct hit.
(DOCX)

File S1 **Appendix S1,** Pseudocode for template matching procedure. **Appendix S2,** Definition of standard statistical terms. **Appendix S3,** Preliminary Logistic Regression Details. **Appendix S4,** Scaling behavior in model systems.
(DOCX)
