# Supplementary figures and images for: Rapid Catalytic Template Searching as an Enzyme Function Prediction Procedure
Source: PLoS One. 2013 May 10;8(5):e62535. doi: 10.1371/journal.pone.0062535 (PMC3651201; doi:10.1371/journal.pone.0062535)

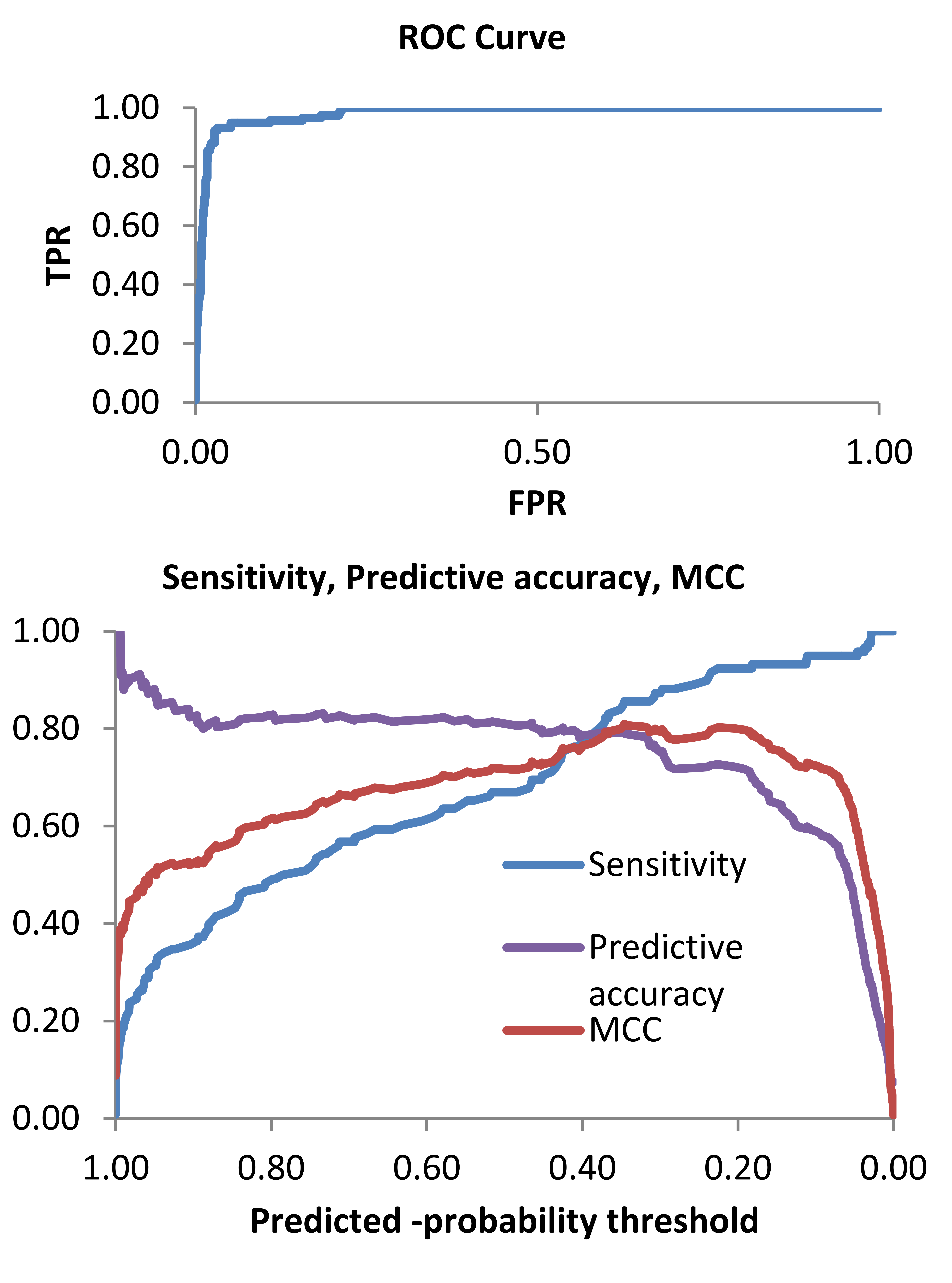

Supplement: Figure S1 — Performance of dataset for comparison to Torrance study. A) Receiver Operating Characteristic b) Sensitivity (TPR), Predictive Accuracy and MCC. (TIF) [file pone.0062535.s001.tif]

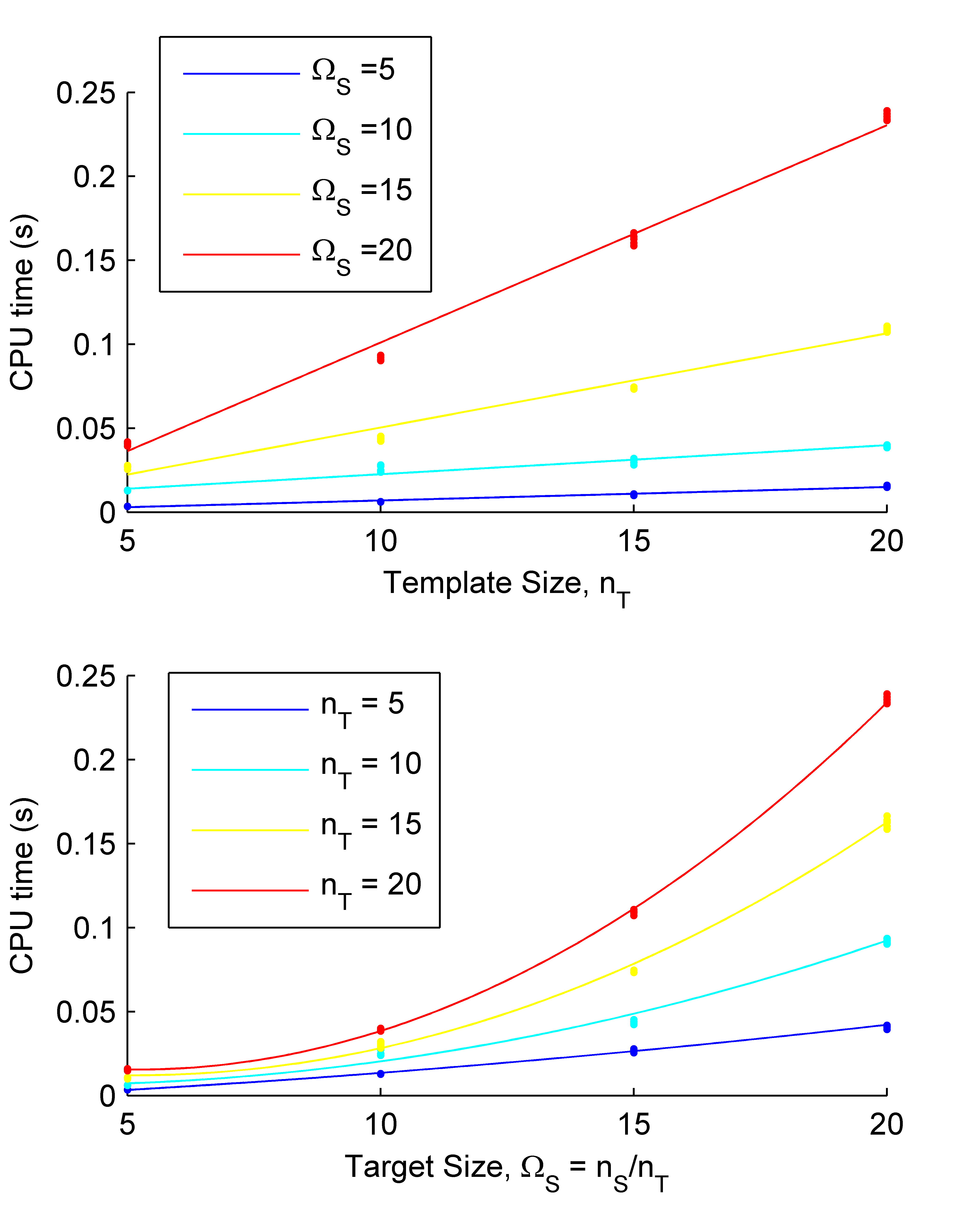

Supplement: Figure S2 — Timings for synthetic template comparisons. Data are shown as points with solid lines as least squares fits. (TIF) [file pone.0062535.s002.tif]
