# Supplementary material for: Rapid Catalytic Template Searching as an Enzyme Function Prediction Procedure
Source: PLoS One. 2013 May 10;8(5):e62535. doi: 10.1371/journal.pone.0062535 (PMC3651201; doi:10.1371/journal.pone.0062535)
Supplement: Table S1 — Targets (member of a template family) and templates – identified by PDB code – included in comparison with JESS. Where the template is not present the algorithm did not return a correct hit. (DOCX) [file pone.0062535.s003.docx]

| **Family member** | **Template** | **Family EC number** |
| --- | --- | --- |
| 1cla | 3cla | 2.03.01.0028 |
| 1cy1 | 1ecl | 5.99.01.0002 |
| 1d6n | 1bzy | 2.04.02.0008 |
| 1dbz |  | 2.07.07.0000 |
| 1dv7 | 1eix | 4.01.01.0023 |
| 1f4c | 1lcb | 2.01.01.0045 |
| 1g02 | 1b2m | 3.01.27.0003 |
| 1gin | 1gim | 6.03.04.0004 |
| 1gzg | 1h7o | 4.02.01.0024 |
| 1hqd | 1tah | 3.01.01.0003 |
| 1hzz | 1l7d | 1.06.01.0002 |
| 1igw |  | 3.02.02.0022 |
| 1kgq | 2tdt | 2.03.01.0117 |
| 1krc |  | 3.02.02.0022 |
| 1l5w | 1gpa | 2.04.01.0001 |
| 1lmz | 1p7m | 3.02.02.0020 |
| 1mj5 | 1cv2 | 3.08.01.0005 |
| 1nwc | 1brm | 1.02.01.0011 |
| 1ojp | 1c82 | 4.02.02.0001 |
| 1p5g | 1p5d | 5.04.02.0008 |
| 1q2e | 1cel | 3.02.01.0091 |
| 1rrj | 1k4t | 5.99.01.0002 |
| 1rsm | 5rsa | 3.01.27.0005 |
| 1rwp | 1qx3 | 3.04.22.0000 |
| 1s70 | 1s95 | 3.01.03.0016 |
| 1t2a | 1db3 | 4.02.01.0047 |
| 1t3z | 1t4c | 2.08.03.0016 |
| 1t4d | 1brm | 1.02.01.0011 |
| 1ucl | 1c54 | 3.01.27.0003 |
| 1w23 | 1bjo | 2.06.01.0052 |
| 1w3y | 1c82 | 4.02.02.0001 |
| 1wo8 | 1b93 | 4.02.03.0003 |
| 1wow | 1dve | 1.14.99.0003 |
| 1wyi | 1hti | 5.03.01.0001 |
| 1ytn | 1ytw | 3.01.03.0048 |
| 1z83 | 1zio | 2.07.04.0003 |
| 1z8x | 1aug | 3.04.19.0003 |
| 2bcd | 1s95 | 3.01.03.0016 |
| 2be7 | 1at1 | 2.01.03.0002 |
| 2brv | 1c82 | 4.02.02.0001 |
| 2g22 | 1bbs | 3.04.23.0015 |
| 2hb1 | 1d5r | 3.01.03.0067 |
| 2ido | 1j53 | 2.07.07.0007 |
| 2j4s | 1bu7 | 1.06.02.0004 |
| 2ori | 1zio | 2.07.04.0003 |
| 2qll | 1gpa | 2.04.01.0001 |
| 2v6s | 1dco | 4.02.01.0096 |
| 2vel | 1tph | 5.03.01.0001 |
| 2vf5 | 1jxa | 2.06.01.0016 |
| 2vmn | 1dfo | 2.01.02.0001 |
| 3cn9 | 1auo | 3.01.01.0001 |
| 3cuf | 1xyz | 3.02.01.0008 |
| 3dt2 | 1nhx | 4.01.01.0032 |
| 3dzc | 1f6d | 5.01.03.0014 |
| 3gxf | 2f61 | 3.02.01.0045 |

Supplementary table 1. Targets (member of a template family) and templates – identified by PDB code – included in comparison with JESS. Where the template is not present the algorithm did not return a correct hit.
