# Supplementary material for: Rapid Catalytic Template Searching as an Enzyme Function Prediction Procedure
Source: PLoS One. 2013 May 10;8(5):e62535. doi: 10.1371/journal.pone.0062535 (PMC3651201; doi:10.1371/journal.pone.0062535)
Supplement: File S1 — Appendix S1, Pseudocode for template matching procedure. Appendix S2, Definition of standard statistical terms. Appendix S3, Preliminary Logistic Regression Details. Appendix S4, Scaling behavior in model systems. (DOCX) [file pone.0062535.s004.docx]

**Appendix S1:** Pseudocode for template matching procedure

The main routine is find_pathlist, with the subroutines and find_adacent_nodes also described append_nodes_to_trial_pathlist.

**function** find_pathlist( T, S ):

***# loop through nodes in template graph***

**for each** node *d* in T (except last node):

***# look for eligible nodes to connect to nodes at site d***

**for each** unique *trailing_node* in ( *pathlist*[*d*][*:*] )

*adjacent_nodes* := find_adjacent_nodes( *trailing_node* )

*# construct a full list of eligible paths with added nodes*

*trial_pathlist* := append_nodes_to_trial_pathlist( *adjacent_nodes* )

**end for**

***# compute subgraph similarities (of size d+1) and ranks them by ms difference***

sort_by_ms_difference( *trial_pathlist* )

***# take top n_path_max hits***

*pathlist*[*1:d*][*:*] = *trial_pathlist* [*:*][*1:n_paths_max*]

clear *trial_pathlist*

**end for**

**end** find_pathlist

The find_adjacent_nodes routine introduces a screen in a similar manner as that outlined by the Ullman algorithm. The present approach constructs this screen iteratively, while the Ullman algorithm stores this in a matrix. We also add the capacity to allow for substitutions by loading a binary substitution matrix. The screen that is applied here is that the distance between residue d and residue d+1 be within some threshold (1.5 Å for the present study) of the template distance between the same set of residues. Note, the distance screen is only applied for residues adjacent in sequence order residue (d,d+1). This formally introduces a dependence on the order of residues in the sequence to be studied (which is alphabetical by 3 letter code and then numerical by residue id in this work). This dependency is easily alleviated by relaxing the number of allowed paths during the search. The present setting is to retain $P_{\max}=50$ paths at every iteration.

**function** *adjacent_nodes* = find_adjacent_nodes( *trailing_node* )

***# storing identity of next node in template***

*template_node.ID* = T(d+1).*ID*

**for each** *structure_node* in S (excluding *trailing_node*)

***# screen for identity (or substitution) and distance similarity***

**if** ( *template_node.ID* == *structure_node.ID* (or *allowed substitution*) and

dist(T( *d* ), T( *d+1* )) – dist(S( *trailing_node* )), S(*structure_node*) ) < *threshold* )

***# constructing allowed nodes for given trailing node***

append *target_node* to *adjacent_nodes*

**end**

**end**

return *adjacent_nodes*

**end** *find_adjacent_nodes*

the append_nodes_to_trial_pathlist routine simply adds the allowed nodes as constructed to a trial_pathlist. The trial_pathlist array is reset at every iteration, and is allocated as the largest array in the routine. For the present study, we found that this was not limiting for any of the cases studied, however.

**function** append_nodes_to_trial_pathlist( *adjacent_nodes* )

***# appending eligible nodes (from adjacent_nodes_list) to trailing_node***

**for each** *path* in *pathlist*[*1:d*][*:*] that contains *trailing_node*:

**for each** *node_to_add* in *adjacent_nodes*

***# screening out nodes that are already present in path***

if *target_node.ID* is **not** in *path*

*path*(*d+1*) := *node_to_add*

append *path*(*1:d+1*) to trial_pathlist

**end**

**end**

**end** append_nodes_to_trial_pathlist

**Appendix S2:** Definition of standard statistical terms

Figures 10-12 make use of standard statistical terms, which are defined below. All terms are defined in terms of 4 fundamental measures, which are the number of true positives $tp$, true negatives$tn$, false positives$fp$, and false negatives$fn$. A true positive is defined as a known positive result that is correctly identified as such according to the procedure of interest. In the present case, the procedure is to apply a cutoff value to a logistic function calculation. The logistic function is given as a multivariate function of the a sum of descriptors,

$f(z)=(1+e^{-z})^{-1}$ Equation S1

Where $f(z)$ is a sigmoidal curve that has a lower bound of zero and upper bound of one. Values close to one predict a positive classification for the observation of interest. Any value above a nominal logistic threshold is thus classified as a positive entry. The total number of entries in a given dataset classified as positive based on this threshold contains a number of entries which are true positives and false positives. A true positive is a result identified by the logistic threshold as positive which is known to be positive. A false positive is predicted as positive while it is actually negative. Likewise, those entries which fall below the threshold are classified as true negatives and false negatives.

The variable $z$ is computed as a simple weighted sum of the descriptors$x_{i}$ and a bias term$\beta_{0}$,

$z=\beta_{0}+\sum_{i} \beta_{i}(n_{T})x_{i}$ Equation S2

A notable difference here is that the$\beta_{i}$ parameters for templates of different size$n_{T}$(or range of sizes) can take on different values, as is described in the Methods section. Table 3 illustrates cases for which template size specific parameters were computed. To estimate these parameters, a dataset is prepared as discussed in the text, with positive and negative annotations, and subjected to a maximum likelihood estimation of parameters using the default settings of the R statistical package.

The total number of known positives in the data set is given as simply$p=tp+fn$, and the number of known negatives is$n=tn+fp$. The true positive rate,$tpr$, is simply a normalized measure of the number of true positives,

$tpr=\frac{tp}{p}$, Equation S3

and the false positive$fpr$rate is given similarly as

$fpr=\frac{fp}{n}$, Equation S4

While there are many measures that can be used to describe the quality of the procedure used, a highly regarded measure is the Matthews Correlation coefficient[[86](#_ENREF_86)], given as

$mcc=\frac{tp\cdot tn-fp\cdot fn}{\sqrt{(tp+fp)\cdot(tn+fn)\cdot p\cdot n}}$ Equation S5

This measure, plotted as a function of threshold, can often guide the choice of optimal threshold value.

An overall measure of performance (over all threshold values) is typically plotted as the receiver operating characteristic curve (ROC). It is simply the true positive rate plotted against the false positive rate. The diagonal line of this plot represents the performance of a random procedure for identifying true positives. Thus, a plot where points lie above the diagonal is considered to be better than random, and a perfect performance would be a step function. To represent this performance, the area under the curve (AUC) metric is computed as the numerical integral computed using the trapezoidal rule:

$AUC=\sum_{t} \frac{1}{2}(tpr_{t+1}+tpr_{t})\cdot(fpr_{t+1}+fpr_{t})$ Equation S6

Where the subscript $t$ is the index of the threshold values for each of the rates.

**Appendix S3:** Preliminary Logistic Regression Details

The preliminary logistic regression was estimated on a smaller, preliminary test set. The preliminary test set consisted of 11 PSIBLAST structure targets and 112 template hits, where those hits were based on a ranking by descriptor 2, the number of distance matrix elements within a threshold value, and descriptor 1, the distance matrix similarity (rmsddm). Backbone rmsd, and SiteMap center (descriptors 5 and 6) were also incorporated, with the difference that the average distance to the SiteMap center was taken (and not the inverse, as describeed in the methods section). At least one “correct hit” – to the LIT template corresponding to each PSIBLAST entry – was included for each target. The 112 hits included 16 correct hits. (There may be more than one correct hit for each target due to “duplicate” binding sites on multi-chain templates and/or duplicate library entries for a given EC number).

**Appendix S4:** Scaling behavior in model systems

Equation 3 and its approximate form suggest that the time to complete a graph search is . This expression assumes that the number of entries $\bar{\Omega}_{S}$ in the target for a particular residue is constant. In actual datasets, this is not true, however, and so we present a timing study on model data that more clearly presents this concept. The data was generated by generating random points within a 20 angstrom sphere and constructing a distance matrix of size$n_{T}\cdot\bar{\Omega}_{S}$, where $n_{T}$ is the template size. Each template is constructed to have no repeating residue ids. To construct the template distance matrix, residues of each (nonrepeating) identity are randomly selected from the target distance matrix to form the submatrix. A Gaussian variate with standard deviation of 0.25 is added to the submatrix to form a slightly mismatched template. This process is repeated 5 times for each parameter setting. To time the process, 100 consecutive runs are carried out, and the resulting time is divided by 100 and reported. Figure S1 shows the resulting times as a function of template size and target size. Note that the scaling with regard to template size appears to be linear, except for in the larger cases. This additional scaling is likely due to additional operations which are beginning to influence the scaling behavior, such as edge construction, sorting, and other operations which are not quantified presently. That the quadratic behavior with respect to distance matrix size is also clearly shown, as was shown in the dataset with the CSA data in Figure 9.
